# Supplementary material for: Palmitoylation of death receptor p75NTR contributes to Alzheimer’s disease progression by regulating APP trafficking and degradation
Source: Alzheimers Res Ther. 2026 Mar 27;18:105. doi: 10.1186/s13195-026-02032-5 (PMC13151129; doi:10.1186/s13195-026-02032-5)

Supplementary figure legends

Supplementary Figure S1. Palmitoylation of PSD95 and caveolin-1 in the hippocampus of 5xFAD mice

ABE assay detection by Western blotting of PSD95 (A) and caveolin-1 (CAV-1) (B) palmitoylation in hippocampus dissected from WT and 5xFAD mice at 2 months of age. Statistical analysis by unpaired t-test; mean ± SEM; N=3 animals per group; *, $p<0.05$.

Supplementary Figure S2. Schematic of strategy for generating palmitoylation-deficient $p75^{C281A}$ mutant mice

Supplementary Figure S3. $p75^{NTR}$ mRNA levels are not affected by the $Cys^{281}Ala$ mutation

Quantitative PCR analysis of $p75^{NTR}$ (*Ngfr*) mRNA levels in the hippocampus of wild type (WT), mutant heterozygous (m/+) and homozygous (m/m) $p75^{NTR}$ mice at postnatal day 3 (P3), P7, and 3 months (3M) of age. Statistical analysis by one-way ANOVA; mean ± SEM; N=3 animals per group at each stage.

Supplementary Figure S4. Dominant effect of palmitoylation-deficient $p75^{NTR}$ on the half-life of wild type $p75^{NTR}$

(A) Lack of palmitoylation does not interefere with $p75^{NTR}$ dimerization. Co-immunoprecipitation between HA-tagged $p75^{NTR}$ (wild type [WT] or palmitoylation-deficient $p75^{NTR}$ mutant [mut]) with Myc-tagged $p75^{NTR}$ (WT or mut) in HEK293T cells that co-transfected with expressing plasmids for 24h. Statistical analysis by one-way ANOVA; mean ± SEM; N=4 independent experiments.

(B) Lack of palmitoylation does not interefere with the palmitoylation of the interacting wild type protomer in the $p75^{NTR}$ dimer. ABE assay analysis of the palmitoylation level of wild type (WT) or palmitoylation-deficient $p75^{NTR}$ mutant (mut) HA-tagged $p75^{NTR}$ when co-expressed with homogenous or heterogenous $p75^{NTR}$ without tag. Histogram shows the relative palmitoylation level of HA-tagged $p75^{NTR}$ (left panel) and all $p75^{NTR}$ (right panel) compared to the condition in which only wild type $p75^{NTR}$ is expressed. Statistical analysis by one-way ANOVA; mean ± SEM; N=3 independent experiments; **$p<0.01$, ***$p<0.001$, ****$p<0.0001$.

(C) Reduced half-life of wild type $p75^{NTR}$ upon co-expression of palmitoylation-deficient mutant. Cycloheximide (CHX) pulse-chase assay of half-life of HA-tagged $p75^{NTR}$ when co-expressed with

wild type (WT) or palmitoylation-deficient mutant p75^{NTR} without tag. Statistical analysis by two-way ANOVA followed by Tukey's multiple comparisons test; mean ± SEM; N=5 independent experiments; ***p<0.001 m/m(m/m) versus WT(WT); #p<0.05, ##p<0.01, ###p<0.001 WT(m/m) versus WT(WT).

Supplementary Figure S5. Wild type and C281A mutant p75^{NTR} interact with 3xFAD APP to a similar extent

Proximity ligation assay (PLA) between p75^{NTR} with 3xFAD-APP in wild type (WT) and m/m hippocampal neurons that infected with AAV-DJ-3xFAD-APP. Histogram shows the numbers of PLA signals per cell. Statistical analysis by unpaired t-test; mean ± SEM; N=3 independent experiments, ≥20 cells per group in each experiment.

Supplementary Figure S6. BACE-1 trafficking is unaffected in mutant neurons

Proximity ligation assay (PLA) between BACE-1 and Rab 5 (A), or Rab7 (B) or Rab11 (C) in primary cultures of hippocampal neurons derived from either wild type (WT) or homozygous mutant (m/m) mice. Histograms show the numbers of PLA signals per cell. Statistical analysis by unpaired t-test; mean ± SEM; N=3 independent experiments, ≥20 cells per group in each experiment.

Supplementary Figure S7. Unchanged APP localization to lipid rafts in hippocampus of 5xFAD mice expressing palmitoylation-deficient p75^{NTR}

(A) Western blot analysis of p75^{NTR} and lipid raft markers caveolin-1 (CAV-1) and ganglioside GM1 in lipid raft fractions 1 to 10 from cultured cortical neurons. Fractions 11 and 12 were excluded due to saturating levels of p75^{NTR} protein. Extremely low levels (<0.1%) of p75^{NTR} protein can be detected in fractions 3 and 4 corresponding to lipid rafts.

(B) Western blot analysis of APP and lipid raft marker caveolin-1 (CAV-1) in lipid raft fractions 1 to 12 from total extracts isolated from the hippocampus of 9 month old 5xFAD mice. Very low levels (<1%) of p75^{NTR} protein can be detected in fractions 5 and 6 corresponding to lipid rafts.

(C) Western blot analysis of APP in lipid raft fractions 5 and 6 (marked by CAV-1) isolated from total extracts of 9 month old 5xFAD mice expressing wild type p75^{NTR} (5xFAD) or palmitoylation-deficient p75^{NTR} (m/m;FAD). Unfractionated total extracts (input) are also shown for comparison. Quantification of results from three independent experiments are shown on the right panel (mean ± SEM).

SUPPLEMENTARY FIGURE. 1

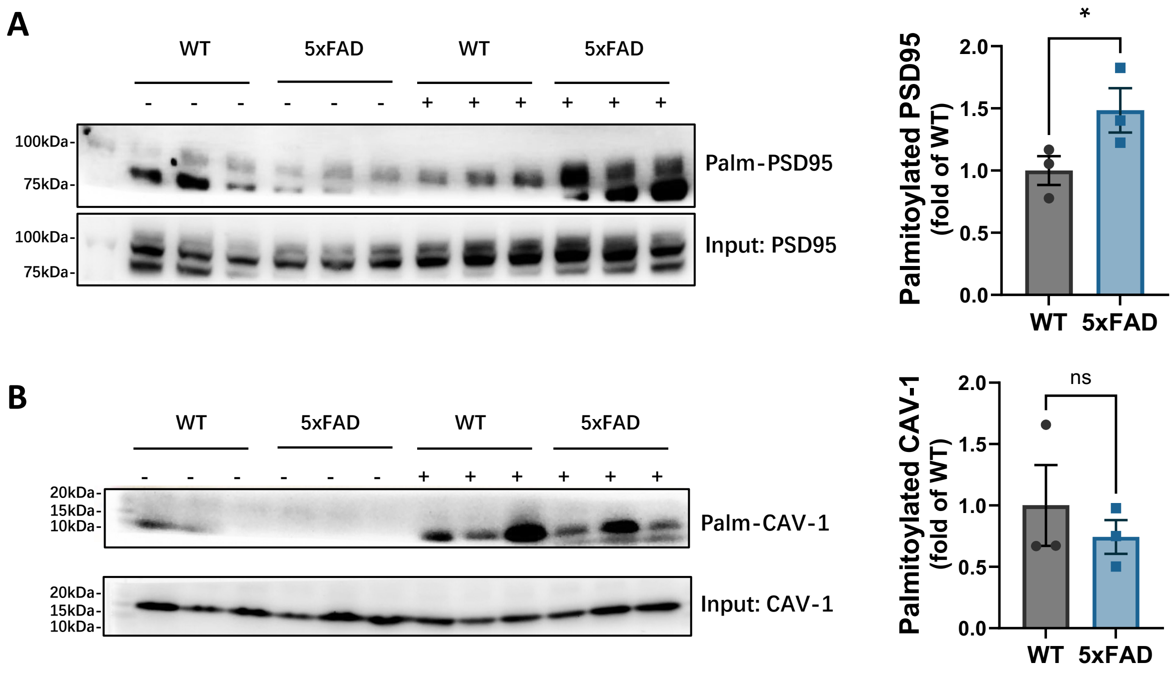

SUPPLEMENTARY FIGURE. 2

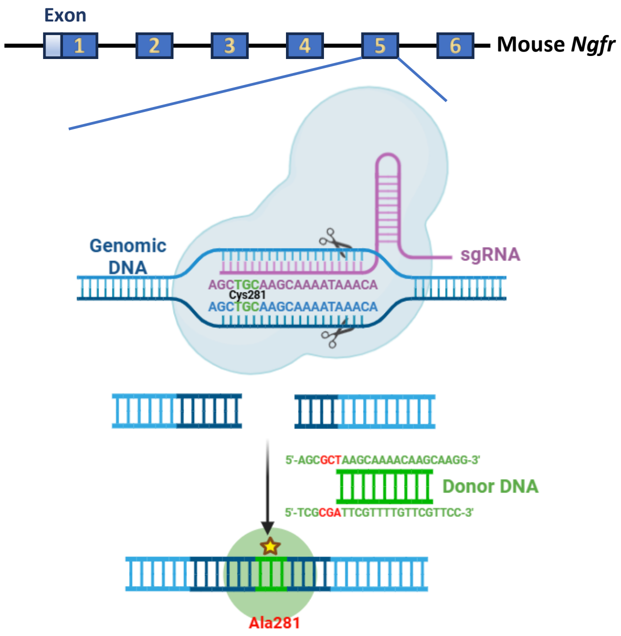

SUPPLEMENTARY FIGURE. 3

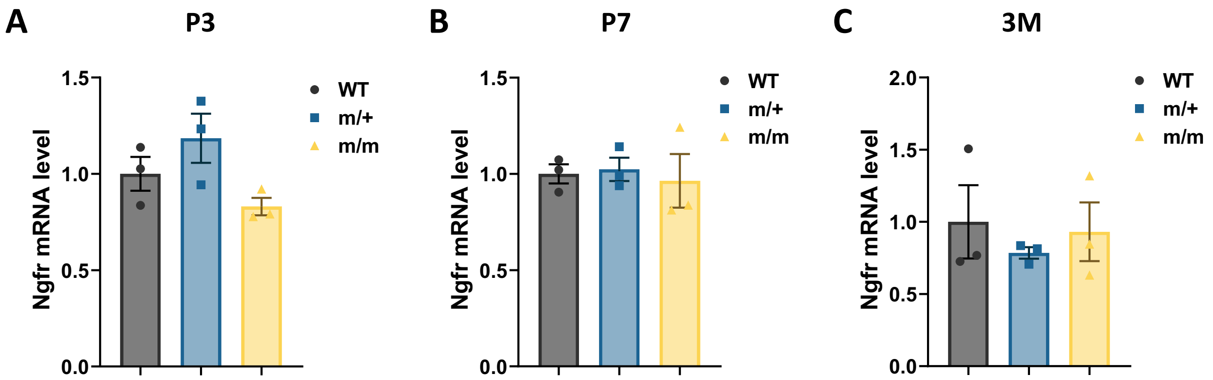

SUPPLEMENTARY FIGURE. 4

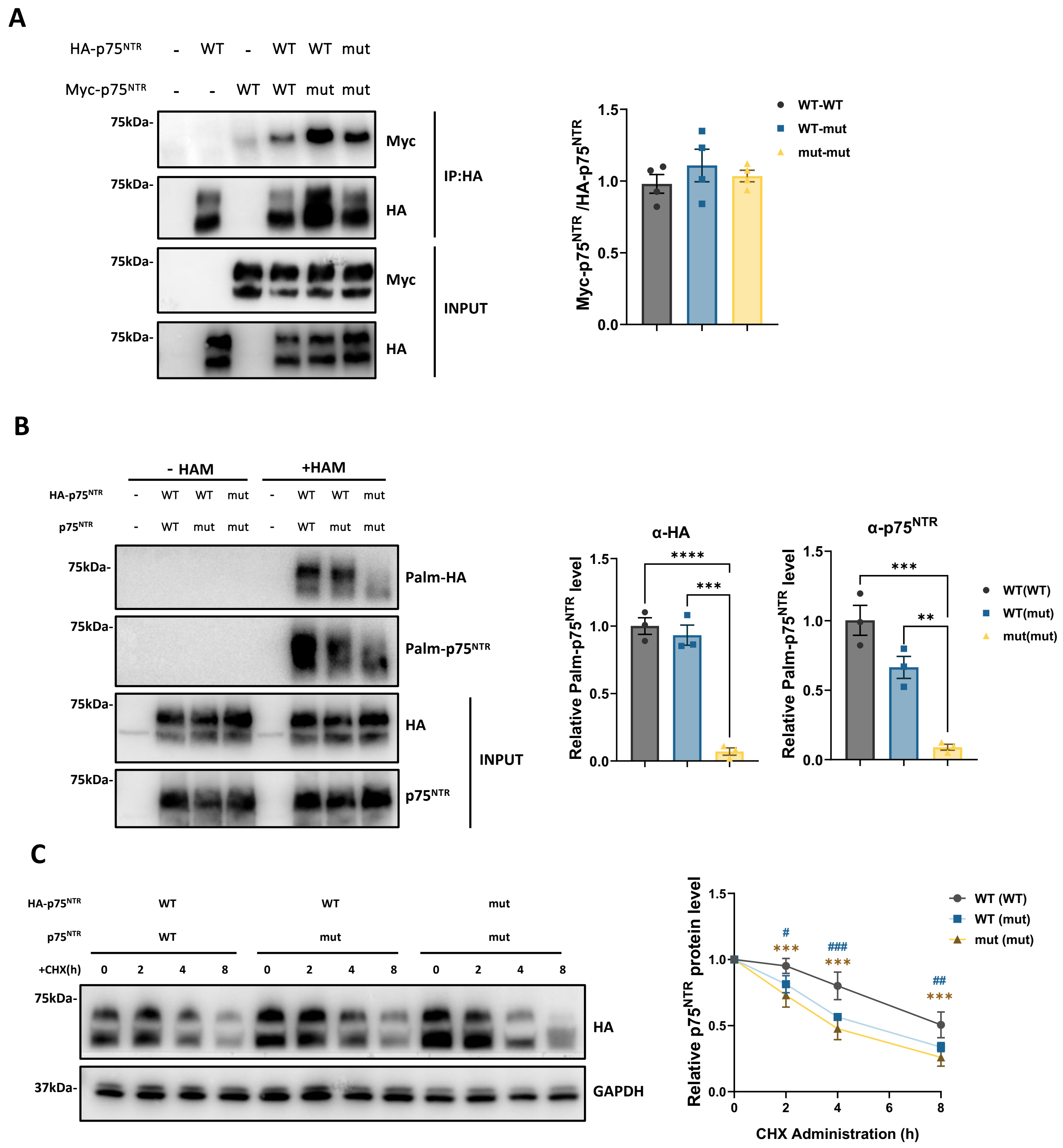

SUPPLEMENTARY FIGURE. 5

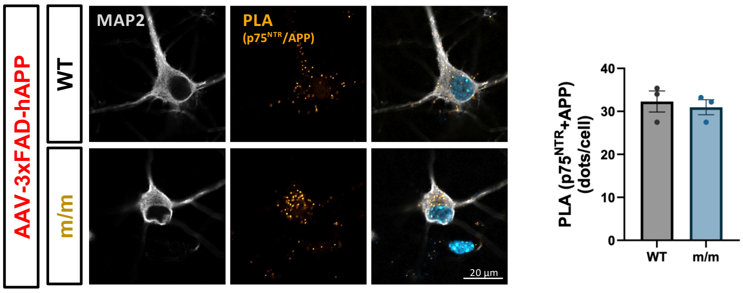

SUPPLEMENTARY FIGURE. 6

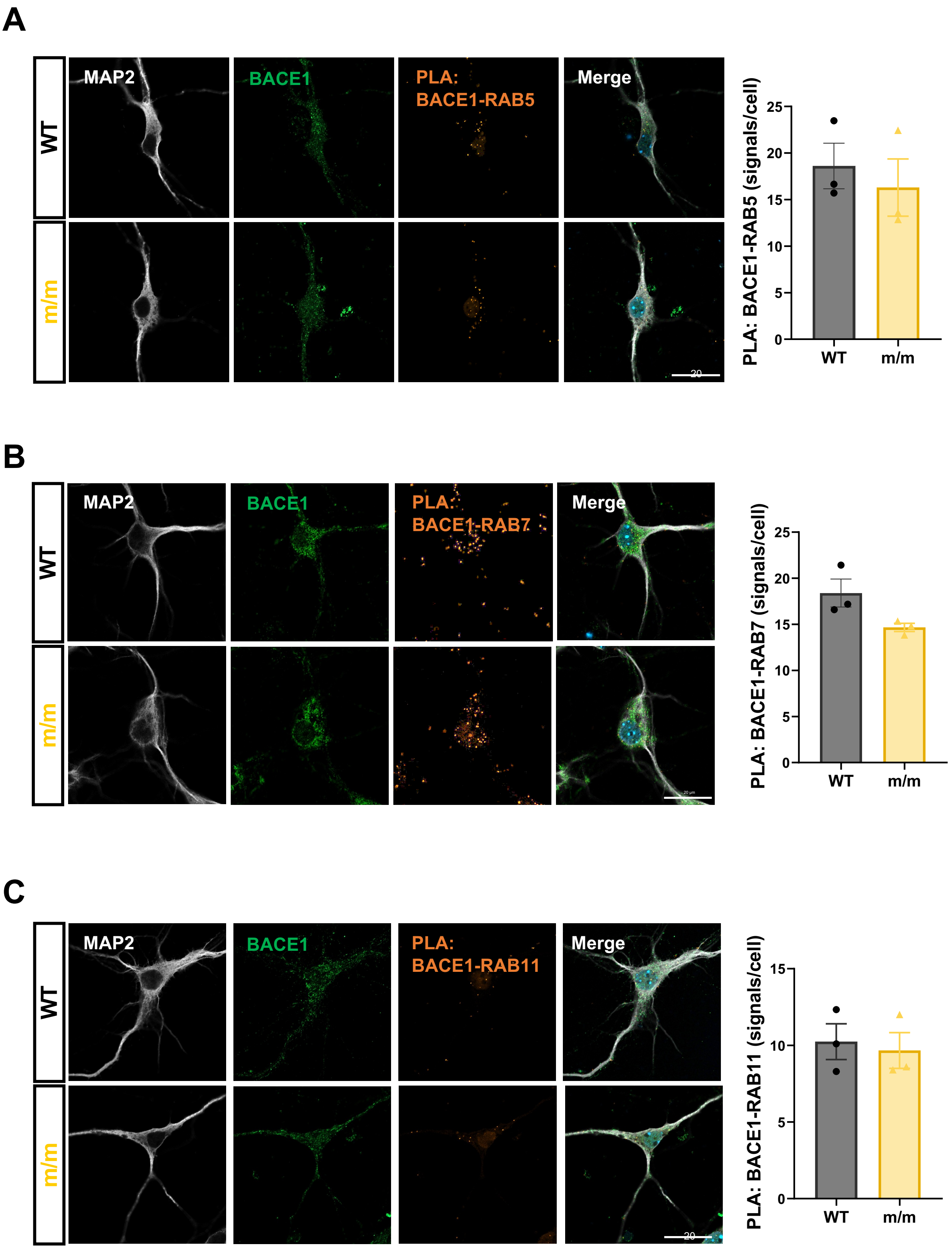

SUPPLEMENTARY FIGURE. 7

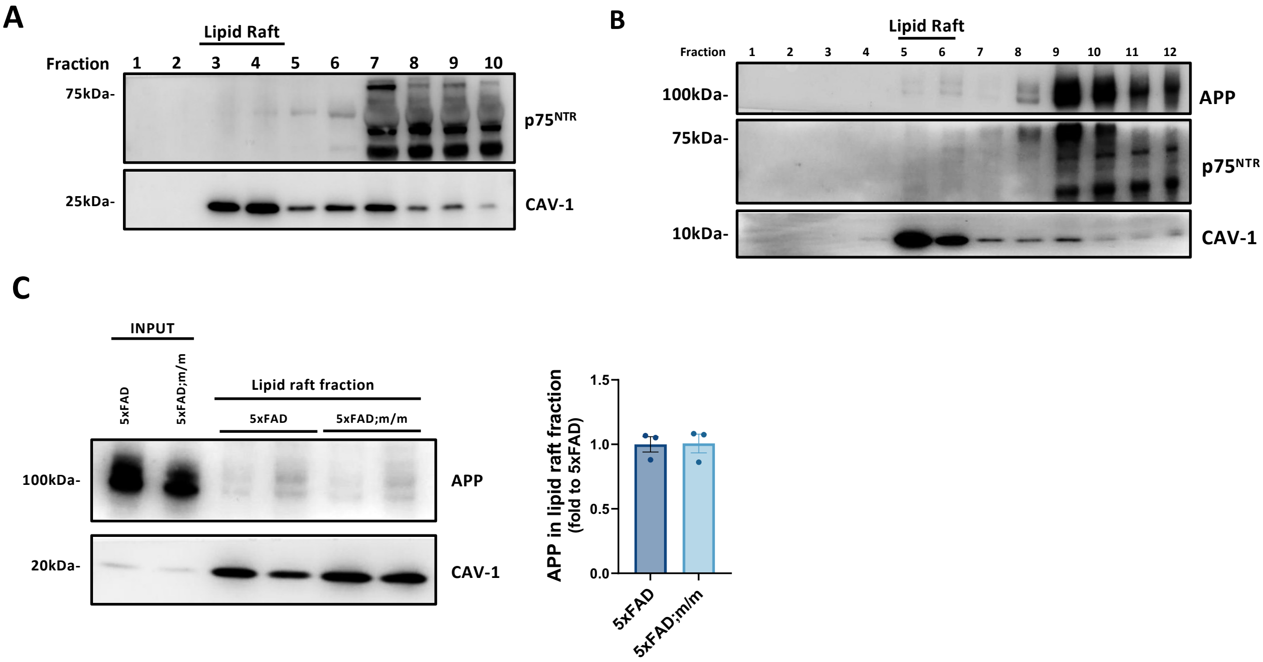

Supplement: Supplementary file 1 — Supplementary Material 1. [file 13195_2026_2032_MOESM1_ESM.pdf]
